# Supplementary material for: Quantized current steps due to the synchronization of microwaves with Bloch oscillations in small Josephson junctions
Source: Nat Commun. 2024 Oct 29;15:9326. doi: 10.1038/s41467-024-53600-y (PMC11522387; doi:10.1038/s41467-024-53600-y)
Supplement: Supplementary file 1 — Supplementary Information [file 41467_2024_53600_MOESM1_ESM.pdf]

Supplementary Information for "Quantised current steps due to the synchronisation of microwaves with the Bloch oscillations in small Josephson junctions"

Rais S. Shaikhaidarov[1,2], Kyung Ho Kim[1], Jacob Dunstan[1], Ilya Antonov[1,2], Dmitry Golubev[3,4], Vladimir N Antonov[1], Oleg V Astafiev[1,5]

[1*]Physics, Royal Holloway University of London, Egham, TW20 0PN, Surrey, UK.
[2]National Physical Laboratory, Hampton Road, Teddington, TW11 0LW, UK.
[3]HQS Quantum Simulations GmbH, Rintheimer Str. 23, Karlsruhe, 76131, Germany.
[4]Department of Applied Physics, QTF Centre of Excellence, Aalto, 610101, Finland.
[5]Skolkovo Institute of Science and Technology, Bolshoy Boulevard 30, Moscow, 121205, Russia.

Supplementary Note 1: Critical voltage in the weak noise limit

Let us calculate the critical voltage in the weak noise limit

$$k_B T \ll eV_C, \tag{1}$$

where k_B is the Boltzamann's constant, T is the effective temperature, characterizing the noise. The critical voltage is

$$V_C = \frac{\pi E_S}{e}, \tag{2}$$

with

$$E_S = \sqrt{\frac{8\eta}{\pi}} E_p e^{-\eta}, \tag{3}$$

where $E_p = \sqrt{8E_J E_C}$ is the plasma energy of the JJ and $\eta = E_p/E_C = \sqrt{8E_J/E_C}$. Therefore,

$$V_C = \frac{\sqrt{8\pi\eta}}{e} E_p e^{-\eta}. \tag{4}$$

Supplementary Note 2: Critical voltage in the strong noise limit

Now we consider the case when the experiment is likely subject to significant noise

$$k_B T \gtrsim eV_C. \tag{1}$$

In this case, the observed value of V_C differs from Eq. (4) due to noise smearing. Indeed, if we assume that the $I-V$ curve is described by Eq. (4) of the main text, then the maximum observed voltage, denoted as V_C^* (analogous to the apparent critical current I_C^*), is

$$V_C^* = \frac{V_C^2}{8R\delta I_T}. \tag{2}$$

Therefore

$$V_C^* = \frac{\pi\eta E_p^2}{e^2 R\delta I_T} e^{-2\eta}. \tag{3}$$

Note that

$$E_C = \frac{e^2}{2(C_J + C_S)}, \tag{4}$$

where C_J is the junction capacitance and C_S is stray capacitance due to the surrounding circuit. We estimate $C_S \sim 1.2\,\text{fF}$ in our devices. Also the Josephson energy is

$$E_J = \frac{\Delta R_Q}{2R_N}, \tag{5}$$

where both C_J and R_N and are scaled with the junction area A_J as

$$C_J = cA_J, \tag{6}$$

$$R_J = \tfrac{r}{A_J}, \tag{7}$$

where $c = 50\,\text{fF/nm}^2$ and r is the normal resistance per unit area of the junction, which is set up by the oxidation condition during the junction fabrication process. For the device shown in the paper $r = R_N \times A_J = 0.6 \times 10^7\,\Omega\text{nm}^2$.

Supplementary Note 3: Environmental circuit

Normal resistors

The normal resistor are part of the protective circuit of the current and voltage probes Fig. 1(a). The resistors are made of Pd film 15 nm thick with $R_\square = 8.8\,\Omega$ at low temperature. To have a compact footprint they are shaped in the form of a meander with width, pitch and length of 150 nm, 350 nm and 107 μm (the meander covers an area of $5 \times 7\,\mu\text{m}^2$). The total resistance is $\sim 6.3\,\text{k}\Omega$ (the meander has 713 squares).

Inductance

The inductors are made of highly disordered 5 nm thick TiN film. They are formed as a meander with a wire width of 100 nm and a pitch of 200 nm, see Fig. 1(a). The meander $L_1 = 1.15\,\mu\text{H}$ has a footprint $5 \times 7\,\mu\text{m}^2$ with total wire length 187 μm (1872 squares). The meander $L_2 = 0.34\,\mu\text{H}$ has a footprint $3 \times 3\,\mu\text{m}^2$ with total wire length 51 μm (512 squares).

Quasi-particle traps

The quasiparticles traps are intended to relax the quasiparticle generated in the TiN meanders when under MW radiation [1]. They are positioned at both sides of the L_1 meander and have size $5\times5\,\mu\text{m}^2$. The traps are a sandwich of 5 nm thick TiN film, 15 nm layer of Pd, and 70 nm layer of Al. The normal metal, Pd, suppresses the superconducting gap of the TiN/Al/Pd stack. Because of the lower superconducting gap in the trap compared to that in pure TiN, the quasiparticles generated in the TiN meanders relax there.

Supplementary Note 4: Current quantization

The sample discussed in the main paper has the current quantization at different frequencies of the MW radiation. The $I-V$ curves with the current plateaus are shown in Supplementary Figure 1(a). The curves are taken under different frequencies and amplitudes of the MWs. Supplementary Figure 1(b) shows the current quantization at different amplitudes of the MWs for a single frequency of 6.975 GHz.

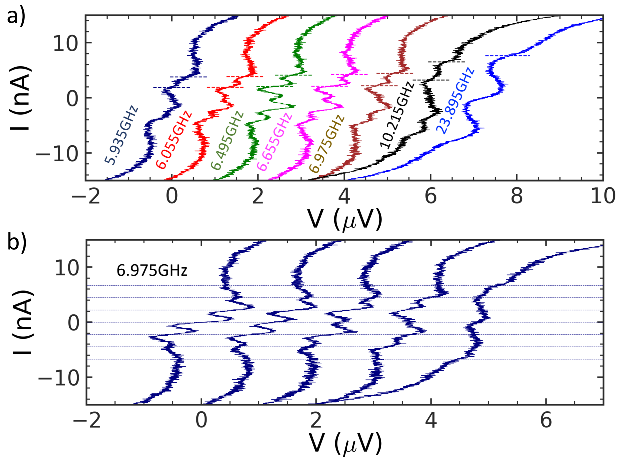

Supplementary Figure 1 (a) $I-V$ curves of one sample under different MW frequencies. The driving amplitude I_{ac} is also different, (b) Appearance of the $I-V$ curve under the fixed MWs of 6.975 GHz with different I_{ac}. The dashed lines indicate the expected position of the quantized current with different m

.

Current quantization has been observed in five samples. Supplementary Figure 2 shows the $I-V$ curves of different samples under the MW. The samples have different V_C^* and I_C^*.

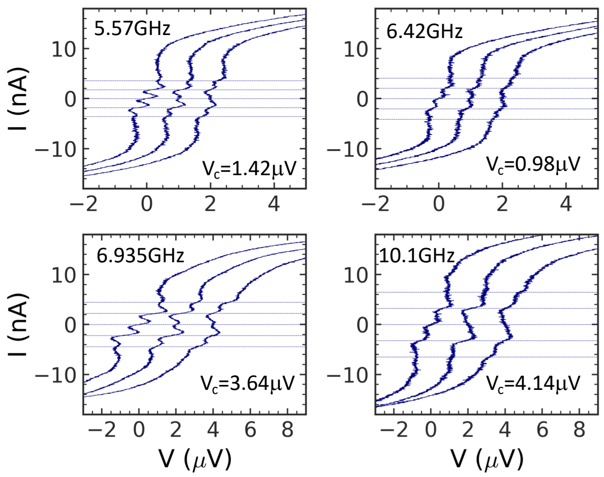

Supplementary Figure 2 Examples of the $I-V$ curves of four samples under different MW frequencies and amplitudes. Each set is measured at fixed frequency with variation of the I_{ac}: $1 < I_{\mathrm{ac}}/(2ef) < 3$. The samples have different apparent critical currents and voltages, I_C^* and V_C^*.

Supplementary Note 5: Modelling dV/dI under the MW radiation

In order to model dV/dI under the MW radiation we take the derivative of Eq. (4) in the main text and obtain the differential resistance at non-zero MW power

$$\frac{dV(I_{\mathrm{dc}})}{dI_{\mathrm{dc}}} = \sum_m J_m^2\left(\frac{I_{\mathrm{ac}}}{2ef}\right)\frac{dV_0(I_{\mathrm{dc}}-2efm)}{dI_{\mathrm{dc}}}. \tag{1}$$

This equation allows one to obtain the differential resistance for any MW power provided the differential resistance $dV(I_{\mathrm{dc}})/dI_{\mathrm{dc}}$ at $I_{\mathrm{ac}}=0$ is known. If we take into account heating of the resistive elements under MW power, then Eq. (1) takes the form [2]:

$$\frac{dV(I_{\mathrm{dc}})}{dI_{\mathrm{dc}}} = \sum_m J_m^2\left(\frac{I_{\mathrm{ac}}}{2ef}\right) A\frac{\Delta I_T^2-(I_{\mathrm{dc}}-2efm)^2}{\left(\Delta I_T^2+(I_{\mathrm{dc}}-2efm)^2\right)^2}. \tag{2}$$

There is an additional fit parameter ΔI_T, which describes the finite dV/dI peak width at zero bias. We adjust ΔI_T for every value of I_{dc} to achieve the best fit to the experimental data. The fitting of a few individual dV/dI traces is shown in Supplementary Figure 3. The modelled curves satisfactory follow the experimental ones of the current plateaus with low $m=0,1,2$. The fits are less accurate at higher m. The simulation allows us to find the absolute value of I_{ac}, which we use in Fig. 3 and 4 of the main text.

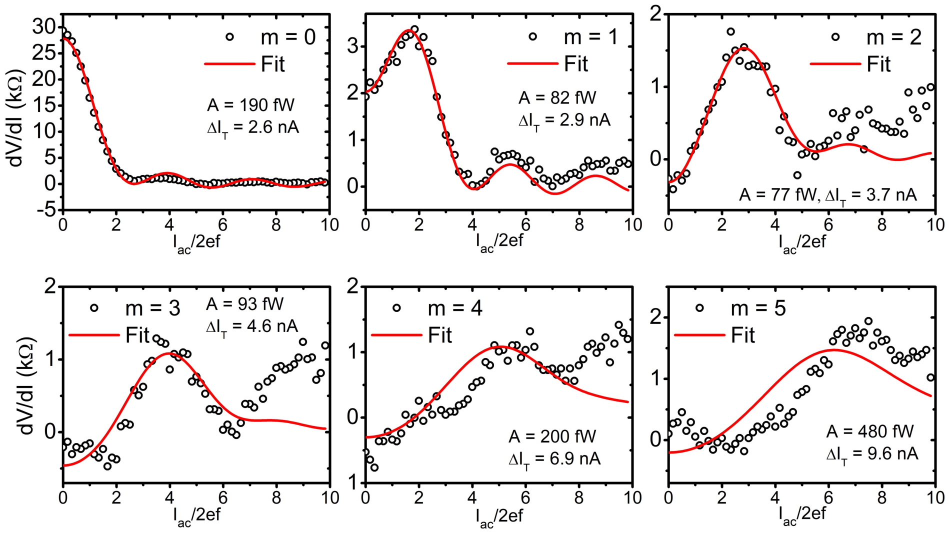

Supplementary Figure 3 dV/dI curves taken under MWs of 6.496 GHz and at $I_{\rm dc}$ corresponding to the quantized current plateau with index m (circles). The red solid line is the fit with the Eq. (2). The width of the dV/dI at zero bias ΔI_T, and the amplitude A are the two fitting parameters.

Supplementary Note 6: Different regimes of oxidation of JJ

All data shown above pertains to a batch of samples subjected to an oxidation pressure of $P_{\rm ox} = 1\,\mu$Bar during the fabrication of the JJs. Additional batches were fabricated under different conditions, as discussed in the paper and summarized in Fig. 5 of the main paper. Supplementary Figure 4 displays examples of $I - V$ and dV/dI characteristics for samples from these varied oxidation conditions: (a-c) for samples fabricated at $P_{\rm ox} = 40\,\mu$Bar; (d-f) for samples fabricated at $P_{\rm ox} = 1\,$mBar.

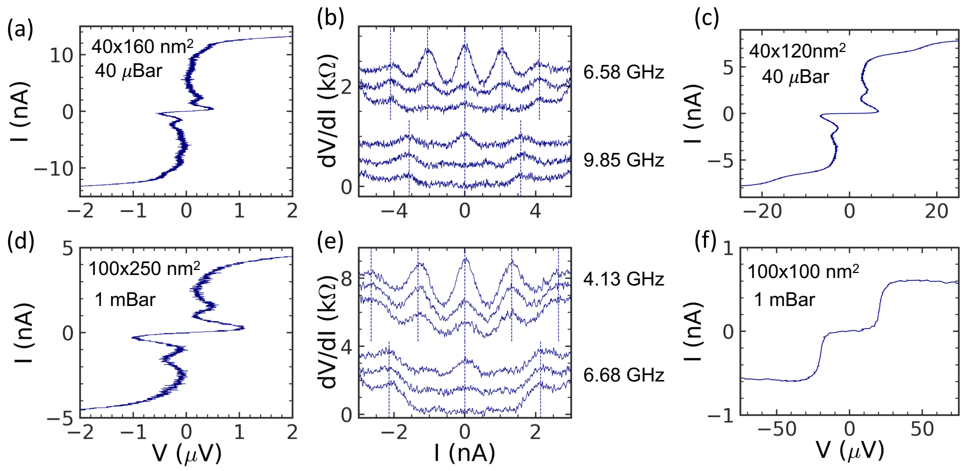

Supplementary Figure 4 (a) Examples of the $I - V$ curve of a sample fabricated at $40\,\mu$Bar oxidation with junction size $40 \times 160\,\mathrm{nm}^2$; (b) Typical dV/dI characteristics of sample (a) at two frequencies of applied MWs and a set of powers; (c) Large blockade of the sample with $40\,\mu$Bar oxidation and junction size $40 \times 100\,\mathrm{nm}^2$. No response to the MWs is observed; (d) $I - V$ curve of a sample with 1 mBar oxidation and junction size $100 \times 250\,\mathrm{nm}^2$; (e) dV/dI characteristics of sample (d) at two frequencies of applied MWs and a set of powers; (f) Large blockade of the sample with 1 mBar oxidation pressure and junction size $100 \times 100\,\mathrm{nm}^2$. No response to the MWs is observed.

Supplementary Note 7: *dc* symmetric amplifier

For the *dc* measurement we use a symmetric bias scheme with three instrumental amplifiers, see Supplementary Figure 5. There are two bias resistor $R_b = 100\,\mathrm{k}\Omega$ in each arm of the scheme. The voltage across the sample is taken at "out1" with an amplification factor G_1, while the current, I, is calculated from the voltage $2G_3R_bI$ at "out3". The scheme operates in the voltage bias regime when sample resistance is high, $R \gg R_b$, and in the current bias regime in the opposite case. At the current plateau we have $R \sim R_b$. This implies that the scheme is in an intermediate regime. It allows us to measure the back bending in the $I - V$ curves.

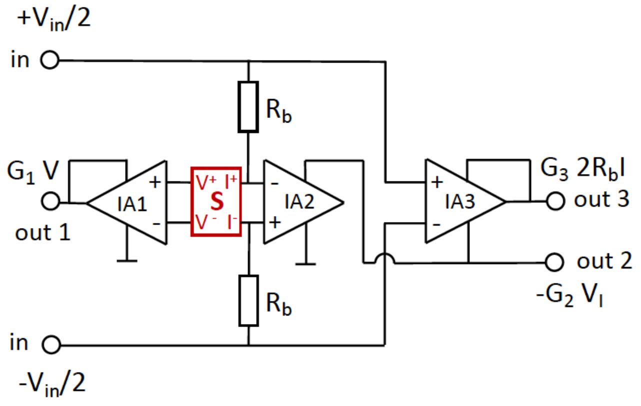

Supplementary Figure 5 The *dc* fully symmetric measurement circuit. The picture of circuit is taken from the Supplementary Information of [2].

Supplementary Note 8: MW transmission characteristics

The transmission of MWs is very non-monotic in frequency. The transmission line together with the environmental circuit has a set of frequencies with strong MW coupling to the JJ. Supplementary Figure 6 shows dV/dI characteristic of zero-bias current blockade as a function of frequency at the output power of generator -5 dBm. The curves show the variation of the MW coupling to the device with frequency with a number of resonances. Although, the current quantization is observed independently of this resonances, it is more convenient to use frequencies close to these resonances, as a weaker MW power ensures reduced heating of the low temperature environment.

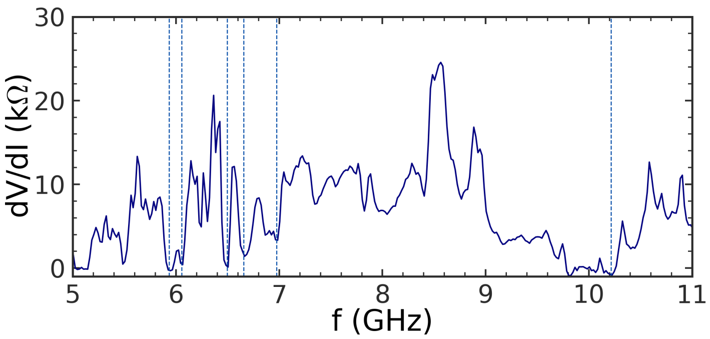

Supplementary Figure 6 Microwave response of a sample to different MW frequencies. The dI/dV peak at zero bias strongly responds to the MW at the resonant frequencies of the circuit. The MW at this frequencies is convenient for observation of the current quantization. The dashed vertical line corresponds to selected frequencies of Supplementary Figure 1.

Supplementary References

[1] Pekola, J.P., Angel, D.V., Suppula, T.I., Suoknuuti, J.K., Manninen, A.J., Manninen, A.: Trapping of quasiparticles of a nonequilibrium superconductor. Appl.Phys.Lett. **76**, 2782 (2000) https://doi.org/10.1063/1.126474

[2] Shaikhaidarov, R.S., Kim, K.H., Dunstan, J.W., Antonov, I.V., Linzen, S., Ziegler, M., Golubev, D.S., Antonov, V.N., Il'ichev, E.V., Astafiev, O.V.: Quantized current steps due to the a.c. coherent quantum phase-slip effect. Nature **608**, 45 (2022) https://doi.org/10.1038/s41586-022-04947-z
